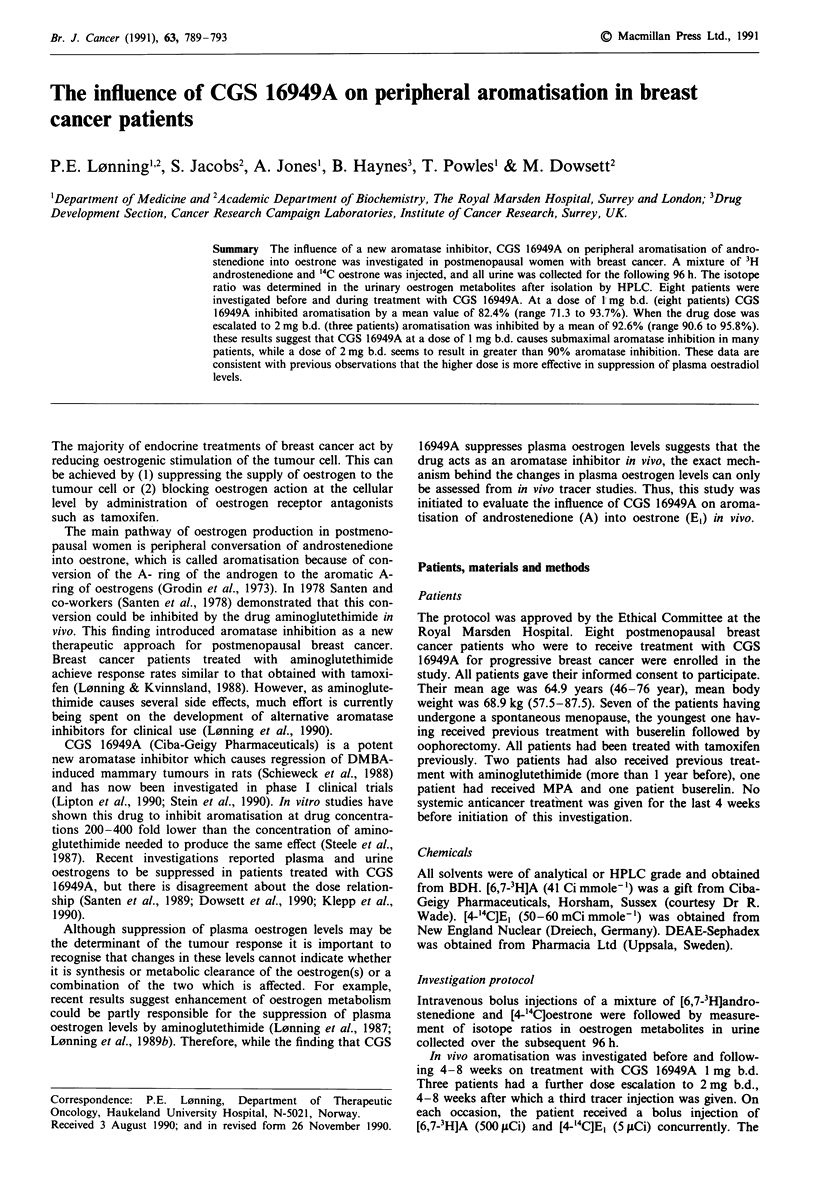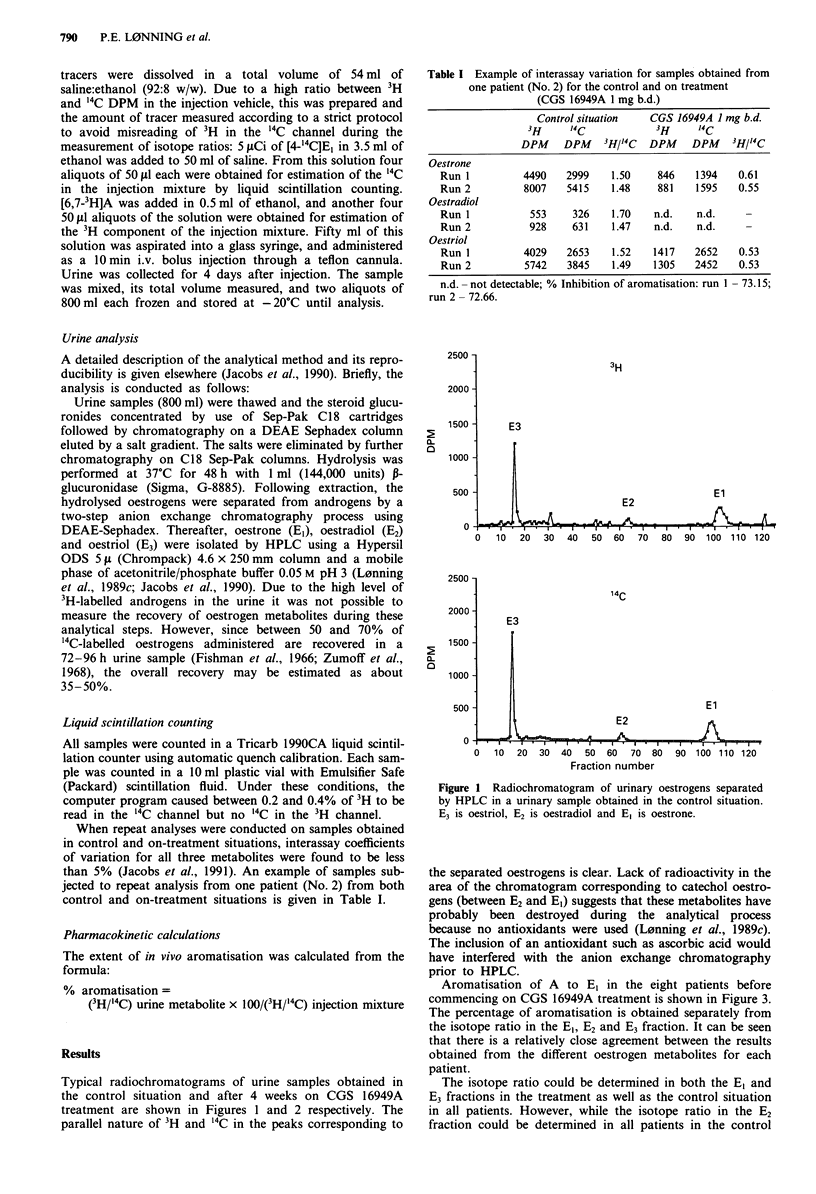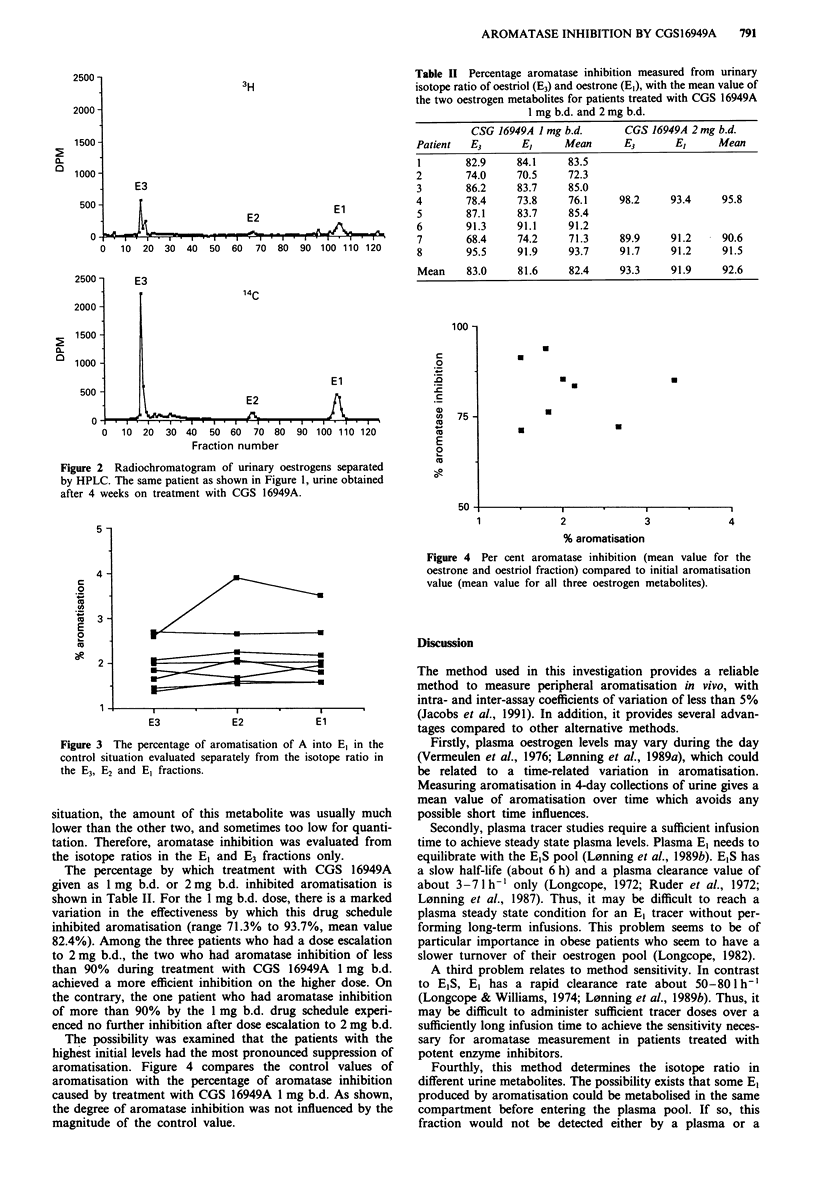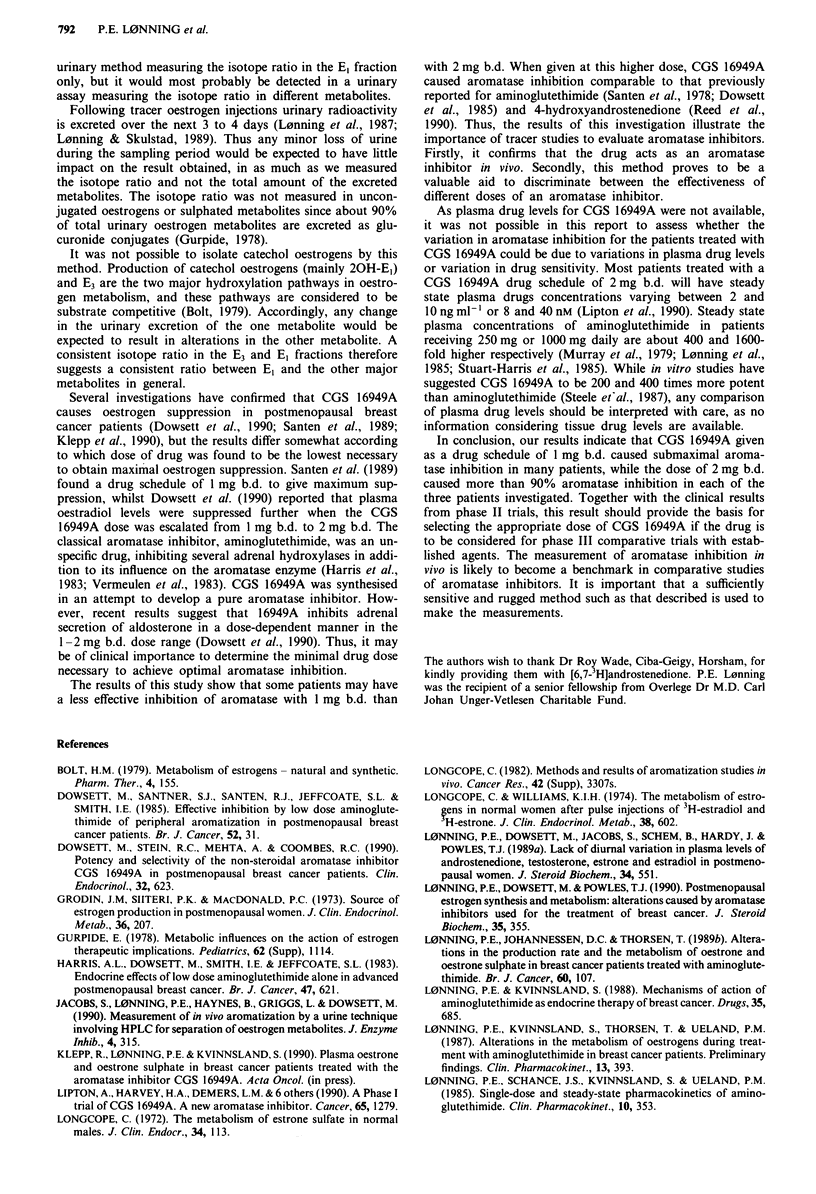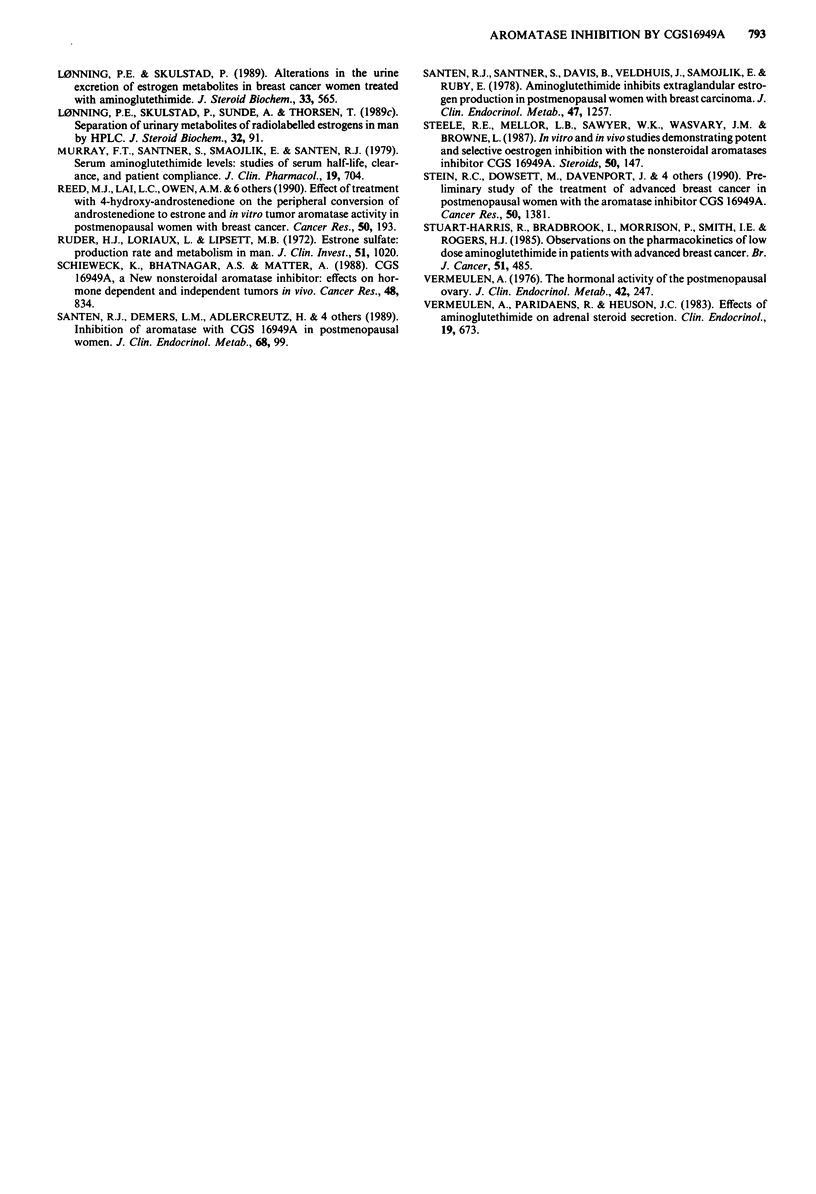# The influence of CGS 16949A on peripheral aromatisation in breast cancer patients.

**DOI:** 10.1038/bjc.1991.175

**Published:** 1991-05

**Authors:** P. E. Lønning, S. Jacobs, A. Jones, B. Haynes, T. Powles, M. Dowsett

**Affiliations:** Department of Medicine, Royal Marsden Hospital, Surrey, UK.

## Abstract

The influence of a new aromatase inhibitor, CGS 16949A on peripheral aromatisation of androstenedione into oestrone was investigated in postmenopausal women with breast cancer. A mixture of 3H androstenedione and 14C oestrone was injected, and all urine was collected for the following 96 h. The isotope ratio was determined in the urinary oestrogen metabolites after isolation by HPLC. Eight patients were investigated before and during treatment with CGS 16949A. At a dose of 1 mg b.d. (eight patients) CGS 16949A inhibited aromatisation by a mean value of 82.4% (range 71.3 to 93.7%). When the drug dose was escalated to 2 mg b.d. (three patients) aromatisation was inhibited by a mean of 92.6% (range 90.6 to 95.8%), these results suggest that CGS 16949A at a dose of 1 mg b.d. causes submaximal aromatase inhibition in many patients, while a dose of 2 mg b.d. seems to result in greater than 90% aromatase inhibition. These data are consistent with previous observations that the higher dose is more effective in suppression of plasma oestradiol levels.


Br. J. Cancer (1991), 63, 789–793 © Macmillan Press Ltd., 1991

### The influence of CGS 16949A on peripheral aromatisation in breast cancer patients

P.E. Lønning[1,2], S. Jacobs[2], A. Jones[1], B. Haynes[3], T. Powles[1] & M. Dowsett[2]

[1]*Department of Medicine and* [2]*Academic Department of Biochemistry, The Royal Marsden Hospital, Surrey and London;* [3]*Drug Development Section, Cancer Research Campaign Laboratories, Institute of Cancer Research, Surrey, UK.*

**Summary** The influence of a new aromatase inhibitor, CGS 16949A on peripheral aromatisation of androstenedione into oestrone was investigated in postmenopausal women with breast cancer. A mixture of $^3H$ androstenedione and $^{14}C$ oestrone was injected, and all urine was collected for the following 96 h. The isotope ratio was determined in the urinary oestrogen metabolites after isolation by HPLC. Eight patients were investigated before and during treatment with CGS 16949A. At a dose of 1 mg b.d. (eight patients) CGS 16949A inhibited aromatisation by a mean value of 82.4% (range 71.3 to 93.7%). When the drug dose was escalated to 2 mg b.d. (three patients) aromatisation was inhibited by a mean of 92.6% (range 90.6 to 95.8%). these results suggest that CGS 16949A at a dose of 1 mg b.d. causes submaximal aromatase inhibition in many patients, while a dose of 2 mg b.d. seems to result in greater than 90% aromatase inhibition. These data are consistent with previous observations that the higher dose is more effective in suppression of plasma oestradiol levels.

The majority of endocrine treatments of breast cancer act by reducing oestrogenic stimulation of the tumour cell. This can be achieved by (1) suppressing the supply of oestrogen to the tumour cell or (2) blocking oestrogen action at the cellular level by administration of oestrogen receptor antagonists such as tamoxifen.

The main pathway of oestrogen production in postmenopausal women is peripheral conversation of androstenedione into oestrone, which is called aromatisation because of conversion of the A- ring of the androgen to the aromatic A-ring of oestrogens (Grodin *et al.*, 1973). In 1978 Santen and co-workers (Santen *et al.*, 1978) demonstrated that this conversion could be inhibited by the drug aminoglutethimide *in vivo*. This finding introduced aromatase inhibition as a new therapeutic approach for postmenopausal breast cancer. Breast cancer patients treated with aminoglutethimide achieve response rates similar to that obtained with tamoxifen (Lønning & Kvinnsland, 1988). However, as aminoglutethimide causes several side effects, much effort is currently being spent on the development of alternative aromatase inhibitors for clinical use (Lønning *et al.*, 1990).

CGS 16949A (Ciba-Geigy Pharmaceuticals) is a potent new aromatase inhibitor which causes regression of DMBA-induced mammary tumours in rats (Schieweck *et al.*, 1988) and has now been investigated in phase I clinical trials (Lipton *et al.*, 1990; Stein *et al.*, 1990). *In vitro* studies have shown this drug to inhibit aromatisation at drug concentrations 200–400 fold lower than the concentration of aminoglutethimide needed to produce the same effect (Steele *et al.*, 1987). Recent investigations reported plasma and urine oestrogens to be suppressed in patients treated with CGS 16949A, but there is disagreement about the dose relationship (Santen *et al.*, 1989; Dowsett *et al.*, 1990; Klepp *et al.*, 1990).

Although suppression of plasma oestrogen levels may be the determinant of the tumour response it is important to recognise that changes in these levels cannot indicate whether it is synthesis or metabolic clearance of the oestrogen(s) or a combination of the two which is affected. For example, recent results suggest enhancement of oestrogen metabolism could be partly responsible for the suppression of plasma oestrogen levels by aminoglutethimide (Lønning *et al.*, 1987; Lønning *et al.*, 1989*b*). Therefore, while the finding that CGS 16949A suppresses plasma oestrogen levels suggests that the drug acts as an aromatase inhibitor *in vivo*, the exact mechanism behind the changes in plasma oestrogen levels can only be assessed from *in vivo* tracer studies. Thus, this study was initiated to evaluate the influence of CGS 16949A on aromatisation of androstenedione (A) into oestrone ($E_1$) *in vivo*.

Correspondence: P.E. Lønning, Department of Therapeutic Oncology, Haukeland University Hospital, N-5021, Norway.
Received 3 August 1990; and in revised form 26 November 1990.

### Patients, materials and methods

### Patients

The protocol was approved by the Ethical Committee at the Royal Marsden Hospital. Eight postmenopausal breast cancer patients who were to receive treatment with CGS 16949A for progressive breast cancer were enrolled in the study. All patients gave their informed consent to participate. Their mean age was 64.9 years (46–76 year), mean body weight was 68.9 kg (57.5–87.5). Seven of the patients having undergone a spontaneous menopause, the youngest one having received previous treatment with buserelin followed by oophorectomy. All patients had been treated with tamoxifen previously. Two patients had also received previous treatment with aminoglutethimide (more than 1 year before), one patient had received MPA and one patient buserelin. No systemic anticancer treatment was given for the last 4 weeks before initiation of this investigation.

### Chemicals

All solvents were of analytical or HPLC grade and obtained from BDH. [6,7-$^3$H]A (41 Ci mmole$^{-1}$) was a gift from Ciba-Geigy Pharmaceuticals, Horsham, Sussex (courtesy Dr R. Wade). [4-$^{14}$C]$E_1$ (50–60 mCi mmole$^{-1}$) was obtained from New England Nuclear (Dreiech, Germany). DEAE-Sephadex was obtained from Pharmacia Ltd (Uppsala, Sweden).

### Investigation protocol

Intravenous bolus injections of a mixture of [6,7-$^3$H]androstenedione and [4-$^{14}$C]oestrone were followed by measurement of isotope ratios in oestrogen metabolites in urine collected over the subsequent 96 h.

*In vivo* aromatisation was investigated before and following 4–8 weeks on treatment with CGS 16949A 1 mg b.d. Three patients had a further dose escalation to 2 mg b.d., 4–8 weeks after which a third tracer injection was given. On each occasion, the patient received a bolus injection of [6,7-$^3$H]A (500 $\mu$Ci) and [4-$^{14}$C]$E_1$ (5 $\mu$Ci) concurrently. The tracers were dissolved in a total volume of 54 ml of saline:ethanol (92:8 w/w). Due to a high ratio between $^{3}H$ and $^{14}C$ DPM in the injection vehicle, this was prepared and the amount of tracer measured according to a strict protocol to avoid misreading of $^{3}H$ in the $^{14}C$ channel during the measurement of isotope ratios: 5 μCi of [4-$^{14}C$]$E_1$ in 3.5 ml of ethanol was added to 50 ml of saline. From this solution four aliquots of 50 μl each were obtained for estimation of the $^{14}C$ in the injection mixture by liquid scintillation counting. [6,7-$^{3}H$]A was added in 0.5 ml of ethanol, and another four 50 μl aliquots of the solution were obtained for estimation of the $^{3}H$ component of the injection mixture. Fifty ml of this solution was aspirated into a glass syringe, and administered as a 10 min i.v. bolus injection through a teflon cannula. Urine was collected for 4 days after injection. The sample was mixed, its total volume measured, and two aliquots of 800 ml each frozen and stored at −20°C until analysis.

*Urine analysis*

A detailed description of the analytical method and its reproducibility is given elsewhere (Jacobs *et al.*, 1990). Briefly, the analysis is conducted as follows:

Urine samples (800 ml) were thawed and the steroid glucuronides concentrated by use of Sep-Pak C18 cartridges followed by chromatography on a DEAE Sephadex column eluted by a salt gradient. The salts were eliminated by further chromatography on C18 Sep-Pak columns. Hydrolysis was performed at 37°C for 48 h with 1 ml (144,000 units) β-glucuronidase (Sigma, G-8885). Following extraction, the hydrolysed oestrogens were separated from androgens by a two-step anion exchange chromatography process using DEAE-Sephadex. Thereafter, oestrone ($E_1$), oestradiol ($E_2$) and oestriol ($E_3$) were isolated by HPLC using a Hypersil ODS 5 μ (Chrompack) 4.6 × 250 mm column and a mobile phase of acetonitrile/phosphate buffer 0.05 M pH 3 (Lønning *et al.*, 1989*c*; Jacobs *et al.*, 1990). Due to the high level of $^{3}H$-labelled androgens in the urine it was not possible to measure the recovery of oestrogen metabolites during these analytical steps. However, since between 50 and 70% of $^{14}C$-labelled oestrogens administered are recovered in a 72–96 h urine sample (Fishman *et al.*, 1966; Zumoff *et al.*, 1968), the overall recovery may be estimated as about 35–50%.

*Liquid scintillation counting*

All samples were counted in a Tricarb 1990CA liquid scintillation counter using automatic quench calibration. Each sample was counted in a 10 ml plastic vial with Emulsifier Safe (Packard) scintillation fluid. Under these conditions, the computer program caused between 0.2 and 0.4% of $^{3}H$ to be read in the $^{14}C$ channel but no $^{14}C$ in the $^{3}H$ channel.

When repeat analyses were conducted on samples obtained in control and on-treatment situations, interassay coefficients of variation for all three metabolites were found to be less than 5% (Jacobs *et al.*, 1991). An example of samples subjected to repeat analysis from one patient (No. 2) from both control and on-treatment situations is given in Table I.

*Pharmacokinetic calculations*

The extent of *in vivo* aromatisation was calculated from the formula:

$$\% \text{ aromatisation} = (^{3}H/^{14}C) \text{ urine metabolite} \times 100/(^{3}H/^{14}C) \text{ injection mixture}$$

**Results**

Typical radiochromatograms of urine samples obtained in the control situation and after 4 weeks on CGS 16949A treatment are shown in Figures 1 and 2 respectively. The parallel nature of $^{3}H$ and $^{14}C$ in the peaks corresponding to the separated oestrogens is clear. Lack of radioactivity in the area of the chromatogram corresponding to catechol oestrogens (between $E_2$ and $E_1$) suggests that these metabolites have probably been destroyed during the analytical process because no antioxidants were used (Lønning *et al.*, 1989*c*). The inclusion of an antioxidant such as ascorbic acid would have interfered with the anion exchange chromatography prior to HPLC.

Aromatisation of A to $E_1$ in the eight patients before commencing on CGS 16949A treatment is shown in Figure 3. The percentage of aromatisation is obtained separately from the isotope ratio in the $E_1$, $E_2$ and $E_3$ fraction. It can be seen that there is a relatively close agreement between the results obtained from the different oestrogen metabolites for each patient.

The isotope ratio could be determined in both the $E_1$ and $E_3$ fractions in the treatment as well as the control situation in all patients. However, while the isotope ratio in the $E_2$ fraction could be determined in all patients in the control

**Table I** Example of interassay variation for samples obtained from one patient (No. 2) for the control and on treatment (CGS 16949A 1 mg b.d.)

| | *Control situation* | | | *CGS 16949A 1 mg b.d.* | | |
|---|---|---|---|---|---|---|
| | *$^{3}H$ DPM* | *$^{14}C$ DPM* | *$^{3}H/^{14}C$* | *$^{3}H$ DPM* | *$^{14}C$ DPM* | *$^{3}H/^{14}C$* |
| *Oestrone* | | | | | | |
| Run 1 | 4490 | 2999 | 1.50 | 846 | 1394 | 0.61 |
| Run 2 | 8007 | 5415 | 1.48 | 881 | 1595 | 0.55 |
| *Oestradiol* | | | | | | |
| Run 1 | 553 | 326 | 1.70 | n.d. | n.d. | – |
| Run 2 | 928 | 631 | 1.47 | n.d. | n.d. | – |
| *Oestriol* | | | | | | |
| Run 1 | 4029 | 2653 | 1.52 | 1417 | 2652 | 0.53 |
| Run 2 | 5742 | 3845 | 1.49 | 1305 | 2452 | 0.53 |

n.d. – not detectable; % Inhibition of aromatisation: run 1 – 73.15; run 2 – 72.66.

**Figure 1** Radiochromatogram of urinary oestrogens separated by HPLC in a urinary sample obtained in the control situation. $E_3$ is oestriol, $E_2$ is oestradiol and $E_1$ is oestrone.

**Figure 2** Radiochromatogram of urinary oestrogens separated by HPLC. The same patient as shown in Figure 1, urine obtained after 4 weeks on treatment with CGS 16949A.

**Figure 3** The percentage of aromatisation of A into $E_1$ in the control situation evaluated separately from the isotope ratio in the $E_3$, $E_2$ and $E_1$ fractions.

situation, the amount of this metabolite was usually much lower than the other two, and sometimes too low for quantitation. Therefore, aromatase inhibition was evaluated from the isotope ratios in the $E_1$ and $E_3$ fractions only.

The percentage by which treatment with CGS 16949A given as 1 mg b.d. or 2 mg b.d. inhibited aromatisation is shown in Table II. For the 1 mg b.d. dose, there is a marked variation in the effectiveness by which this drug schedule inhibited aromatisation (range 71.3% to 93.7%, mean value 82.4%). Among the three patients who had a dose escalation to 2 mg b.d., the two who had aromatase inhibition of less than 90% during treatment with CGS 16949A 1 mg b.d. achieved a more efficient inhibition on the higher dose. On the contrary, the one patient who had aromatase inhibition of more than 90% by the 1 mg b.d. drug schedule experienced no further inhibition after dose escalation to 2 mg b.d.

The possibility was examined that the patients with the highest initial levels had the most pronounced suppression of aromatisation. Figure 4 compares the control values of aromatisation with the percentage of aromatase inhibition caused by treatment with CGS 16949A 1 mg b.d. As shown, the degree of aromatase inhibition was not influenced by the magnitude of the control value.

**Table II** Percentage aromatase inhibition measured from urinary isotope ratio of oestriol ($E_3$) and oestrone ($E_1$), with the mean value of the two oestrogen metabolites for patients treated with CGS 16949A 1 mg b.d. and 2 mg b.d.

| | *CSG 16949A 1 mg b.d.* | | | *CGS 16949A 2 mg b.d.* | | |
|---|---|---|---|---|---|---|
| *Patient* | $E_3$ | $E_1$ | *Mean* | $E_3$ | $E_1$ | *Mean* |
| 1 | 82.9 | 84.1 | 83.5 | | | |
| 2 | 74.0 | 70.5 | 72.3 | | | |
| 3 | 86.2 | 83.7 | 85.0 | | | |
| 4 | 78.4 | 73.8 | 76.1 | 98.2 | 93.4 | 95.8 |
| 5 | 87.1 | 83.7 | 85.4 | | | |
| 6 | 91.3 | 91.1 | 91.2 | | | |
| 7 | 68.4 | 74.2 | 71.3 | 89.9 | 91.2 | 90.6 |
| 8 | 95.5 | 91.9 | 93.7 | 91.7 | 91.2 | 91.5 |
| Mean | 83.0 | 81.6 | 82.4 | 93.3 | 91.9 | 92.6 |

**Figure 4** Per cent aromatase inhibition (mean value for the oestrone and oestriol fraction) compared to initial aromatisation value (mean value for all three oestrogen metabolites).

### Discussion

The method used in this investigation provides a reliable method to measure peripheral aromatisation *in vivo*, with intra- and inter-assay coefficients of variation of less than 5% (Jacobs *et al.*, 1991). In addition, it provides several advantages compared to other alternative methods.

Firstly, plasma oestrogen levels may vary during the day (Vermeulen *et al.*, 1976; Lønning *et al.*, 1989*a*), which could be related to a time-related variation in aromatisation. Measuring aromatisation in 4-day collections of urine gives a mean value of aromatisation over time which avoids any possible short time influences.

Secondly, plasma tracer studies require a sufficient infusion time to achieve steady state plasma levels. Plasma $E_1$ needs to equilibrate with the $E_1S$ pool (Lønning *et al.*, 1989*b*). $E_1S$ has a slow half-life (about 6 h) and a plasma clearance value of about $3-7\,l\,h^{-1}$ only (Longcope, 1972; Ruder *et al.*, 1972; Lønning *et al.*, 1987). Thus, it may be difficult to reach a plasma steady state condition for an $E_1$ tracer without performing long-term infusions. This problem seems to be of particular importance in obese patients who seem to have a slower turnover of their oestrogen pool (Longcope, 1982).

A third problem relates to method sensitivity. In contrast to $E_1S$, $E_1$ has a rapid clearance rate about $50-80\,l\,h^{-1}$ (Longcope & Williams, 1974; Lønning *et al.*, 1989*b*). Thus, it may be difficult to administer sufficient tracer doses over a sufficiently long infusion time to achieve the sensitivity necessary for aromatase measurement in patients treated with potent enzyme inhibitors.

Fourthly, this method determines the isotope ratio in different urine metabolites. The possibility exists that some $E_1$ produced by aromatisation could be metabolised in the same compartment before entering the plasma pool. If so, this fraction would not be detected either by a plasma or a urinary method measuring the isotope ratio in the $E_1$ fraction only, but it would most probably be detected in a urinary assay measuring the isotope ratio in different metabolites.

Following tracer oestrogen injections urinary radioactivity is excreted over the next 3 to 4 days (Lønning et al., 1987; Lønning & Skulstad, 1989). Thus any minor loss of urine during the sampling period would be expected to have little impact on the result obtained, in as much as we measured the isotope ratio and not the total amount of the excreted metabolites. The isotope ratio was not measured in unconjugated oestrogens or sulphated metabolites since about 90% of total urinary oestrogen metabolites are excreted as glucuronide conjugates (Gurpide, 1978).

It was not possible to isolate catechol oestrogens by this method. Production of catechol oestrogens (mainly 2OH-$E_1$) and $E_3$ are the two major hydroxylation pathways in oestrogen metabolism, and these pathways are considered to be substrate competitive (Bolt, 1979). Accordingly, any change in the urinary excretion of the one metabolite would be expected to result in alterations in the other metabolite. A consistent isotope ratio in the $E_3$ and $E_1$ fractions therefore suggests a consistent ratio between $E_1$ and the other major metabolites in general.

Several investigations have confirmed that CGS 16949A causes oestrogen suppression in postmenopausal breast cancer patients (Dowsett et al., 1990; Santen et al., 1989; Klepp et al., 1990), but the results differ somewhat according to which dose of drug was found to be the lowest necessary to obtain maximal oestrogen suppression. Santen et al. (1989) found a drug schedule of 1 mg b.d. to give maximum suppression, whilst Dowsett et al. (1990) reported that plasma oestradiol levels were suppressed further when the CGS 16949A dose was escalated from 1 mg b.d. to 2 mg b.d. The classical aromatase inhibitor, aminoglutethimide, was an unspecific drug, inhibiting several adrenal hydroxylases in addition to its influence on the aromatase enzyme (Harris et al., 1983; Vermeulen et al., 1983). CGS 16949A was synthesised in an attempt to develop a pure aromatase inhibitor. However, recent results suggest that 16949A inhibits adrenal secretion of aldosterone in a dose-dependent manner in the 1–2 mg b.d. dose range (Dowsett et al., 1990). Thus, it may be of clinical importance to determine the minimal drug dose necessary to achieve optimal aromatase inhibition.

The results of this study show that some patients may have a less effective inhibition of aromatase with 1 mg b.d. than with 2 mg b.d. When given at this higher dose, CGS 16949A caused aromatase inhibition comparable to that previously reported for aminoglutethimide (Santen et al., 1978; Dowsett et al., 1985) and 4-hydroxyandrostenedione (Reed et al., 1990). Thus, the results of this investigation illustrate the importance of tracer studies to evaluate aromatase inhibitors. Firstly, it confirms that the drug acts as an aromatase inhibitor *in vivo*. Secondly, this method proves to be a valuable aid to discriminate between the effectiveness of different doses of an aromatase inhibitor.

As plasma drug levels for CGS 16949A were not available, it was not possible in this report to assess whether the variation in aromatase inhibition for the patients treated with CGS 16949A could be due to variations in plasma drug levels or variation in drug sensitivity. Most patients treated with a CGS 16949A drug schedule of 2 mg b.d. will have steady state plasma drugs concentrations varying between 2 and 10 ng $ml^{-1}$ or 8 and 40 nM (Lipton et al., 1990). Steady state plasma concentrations of aminoglutethimide in patients receiving 250 mg or 1000 mg daily are about 400 and 1600-fold higher respectively (Murray et al., 1979; Lønning et al., 1985; Stuart-Harris et al., 1985). While *in vitro* studies have suggested CGS 16949A to be 200 and 400 times more potent than aminoglutethimide (Steele et al., 1987), any comparison of plasma drug levels should be interpreted with care, as no information considering tissue drug levels are available.

In conclusion, our results indicate that CGS 16949A given as a drug schedule of 1 mg b.d. caused submaximal aromatase inhibition in many patients, while the dose of 2 mg b.d. caused more than 90% aromatase inhibition in each of the three patients investigated. Together with the clinical results from phase II trials, this result should provide the basis for selecting the appropriate dose of CGS 16949A if the drug is to be considered for phase III comparative trials with established agents. The measurement of aromatase inhibition *in vivo* is likely to become a benchmark in comparative studies of aromatase inhibitors. It is important that a sufficiently sensitive and rugged method such as that described is used to make the measurements.

The authors wish to thank Dr Roy Wade, Ciba-Geigy, Horsham, for kindly providing them with [6,7-$^3$H]androstenedione. P.E. Lønning was the recipient of a senior fellowship from Overlege Dr M.D. Carl Johan Unger-Vetlesen Charitable Fund.

### References

BOLT, H.M. (1979). Metabolism of estrogens – natural and synthetic. *Pharm. Ther.*, **4**, 155.

DOWSETT, M., SANTNER, S.J., SANTEN, R.J., JEFFCOATE, S.L. & SMITH, I.E. (1985). Effective inhibition by low dose aminoglutethimide of peripheral aromatization in postmenopausal breast cancer patients. *Br. J. Cancer*, **52**, 31.

DOWSETT, M., STEIN, R.C., MEHTA, A. & COOMBES, R.C. (1990). Potency and selectivity of the non-steroidal aromatase inhibitor CGS 16949A in postmenopausal breast cancer patients. *Clin. Endocrinol.*, **32**, 623.

GRODIN, J.M, SIITERI, P.K. & MACDONALD, P.C. (1973). Source of estrogen production in postmenopausal women. *J. Clin. Endocrinol. Metab.*, **36**, 207.

GURPIDE, E. (1978). Metabolic influences on the action of estrogen therapeutic implications. *Pediatrics*, **62** (Supp), 1114.

HARRIS, A.L., DOWSETT, M., SMITH, I.E. & JEFFCOATE, S.L. (1983). Endocrine effects of low dose aminoglutethimide alone in advanced postmenopausal breast cancer. *Br. J. Cancer*, **47**, 621.

JACOBS, S., LØNNING, P.E., HAYNES, B., GRIGGS, L. & DOWSETT, M. (1990). Measurement of *in vivo* aromatization by a urine technique involving HPLC for separation of oestrogen metabolites. *J. Enzyme Inhib.*, **4**, 315.

KLEPP, R., LØNNING, P.E. & KVINNSLAND, S. (1990). Plasma oestrone and oestrone sulphate in breast cancer patients treated with the aromatase inhibitor CGS 16949A. *Acta Oncol.* (in press).

LIPTON, A., HARVEY, H.A., DEMERS, L.M. & 6 others (1990). A Phase I trial of CGS 16949A. A new aromatase inhibitor. *Cancer*, **65**, 1279.

LONGCOPE, C. (1972). The metabolism of estrone sulfate in normal males. *J. Clin. Endocr.*, **34**, 113.

LONGCOPE, C. (1982). Methods and results of aromatization studies *in vivo*. *Cancer Res.*, **42** (Supp), 3307s.

LONGCOPE, C. & WILLIAMS, K.I.H. (1974). The metabolism of estrogens in normal women after pulse injections of $^3$H-estradiol and $^3$H-estrone. *J. Clin. Endocrinol. Metab.*, **38**, 602.

LØNNING, P.E., DOWSETT, M., JACOBS, S., SCHEM, B., HARDY, J. & POWLES, T.J. (1989*a*). Lack of diurnal variation in plasma levels of androstenedione, testosterone, estrone and estradiol in postmenopausal women. *J. Steroid Biochem.*, **34**, 551.

LØNNING, P.E., DOWSETT, M. & POWLES, T.J. (1990). Postmenopausal estrogen synthesis and metabolism: alterations caused by aromatase inhibitors used for the treatment of breast cancer. *J. Steroid Biochem.*, **35**, 355.

LØNNING, P.E., JOHANNESSEN, D.C. & THORSEN, T. (1989*b*). Alterations in the production rate and the metabolism of oestrone and oestrone sulphate in breast cancer patients treated with aminoglutethimide. *Br. J. Cancer*, **60**, 107.

LØNNING, P.E. & KVINNSLAND, S. (1988). Mechanisms of action of aminoglutethimide as endocrine therapy of breast cancer. *Drugs*, **35**, 685.

LØNNING, P.E., KVINNSLAND, S., THORSEN, T. & UELAND, P.M. (1987). Alterations in the metabolism of oestrogens during treatment with aminoglutethimide in breast cancer patients. Preliminary findings. *Clin. Pharmacokinet.*, **13**, 393.

LØNNING, P.E., SCHANCE, J.S., KVINNSLAND, S. & UELAND, P.M. (1985). Single-dose and steady-state pharmacokinetics of aminoglutethimide. *Clin. Pharmacokinet.*, **10**, 353.

LØNNING, P.E. & SKULSTAD, P. (1989). Alterations in the urine excretion of estrogen metabolites in breast cancer women treated with aminoglutethimide. *J. Steroid Biochem.*, **33**, 565.

LØNNING, P.E., SKULSTAD, P., SUNDE, A. & THORSEN, T. (1989*c*). Separation of urinary metabolites of radiolabelled estrogens in man by HPLC. *J. Steroid Biochem.*, **32**, 91.

MURRAY, F.T., SANTNER, S., SMAOJLIK, E. & SANTEN, R.J. (1979). Serum aminoglutethimide levels: studies of serum half-life, clearance, and patient compliance. *J. Clin. Pharmacol.*, **19**, 704.

REED, M.J., LAI, L.C., OWEN, A.M. & 6 others (1990). Effect of treatment with 4-hydroxy-androstenedione on the peripheral conversion of androstenedione to estrone and *in vitro* tumor aromatase activity in postmenopausal women with breast cancer. *Cancer Res.*, **50**, 193.

RUDER, H.J., LORIAUX, L. & LIPSETT, M.B. (1972). Estrone sulfate: production rate and metabolism in man. *J. Clin. Invest.*, **51**, 1020.

SCHIEWECK, K., BHATNAGAR, A.S. & MATTER, A. (1988). CGS 16949A, a New nonsteroidal aromatase inhibitor: effects on hormone dependent and independent tumors *in vivo*. *Cancer Res.*, **48**, 834.

SANTEN, R.J., DEMERS, L.M., ADLERCREUTZ, H. & 4 others (1989). Inhibition of aromatase with CGS 16949A in postmenopausal women. *J. Clin. Endocrinol. Metab.*, **68**, 99.

SANTEN, R.J., SANTNER, S., DAVIS, B., VELDHUIS, J., SAMOJLIK, E. & RUBY, E. (1978). Aminoglutethimide inhibits extraglandular estrogen production in postmenopausal women with breast carcinoma. *J. Clin. Endocrinol. Metab.*, **47**, 1257.

STEELE, R.E., MELLOR, L.B., SAWYER, W.K., WASVARY, J.M. & BROWNE, L. (1987). *In vitro* and *in vivo* studies demonstrating potent and selective oestrogen inhibition with the nonsteroidal aromatases inhibitor CGS 16949A. *Steroids*, **50**, 147.

STEIN, R.C., DOWSETT, M., DAVENPORT, J. & 4 others (1990). Preliminary study of the treatment of advanced breast cancer in postmenopausal women with the aromatase inhibitor CGS 16949A. *Cancer Res.*, **50**, 1381.

STUART-HARRIS, R., BRADBROOK, I., MORRISON, P., SMITH, I.E. & ROGERS, H.J. (1985). Observations on the pharmacokinetics of low dose aminoglutethimide in patients with advanced breast cancer. *Br. J. Cancer*, **51**, 485.

VERMEULEN, A. (1976). The hormonal activity of the postmenopausal ovary. *J. Clin. Endocrinol. Metab.*, **42**, 247.

VERMEULEN, A., PARIDAENS, R. & HEUSON, J.C. (1983). Effects of aminoglutethimide on adrenal steroid secretion. *Clin. Endocrinol.*, **19**, 673.